# Studies of Mercaptosuccinic Acid-Crosslinked Chitosan Hydrogel with Grafted Cinnamaldehyde and Silver Nanoparticles for Antibacterial Biomedical Application

**DOI:** 10.3390/ijms232314806

**Published:** 2022-11-26

**Authors:** Chi-Hui Cheng, Yao-Yuan Tu, Jui-Che Lin

**Affiliations:** 1 Department of Pediatrics, College of Medicine, Chang Gung University, Chang Gung Memorial Hospital, Taoyuan 33305, Taiwan; 2 Department of Chemical Engineering, National Cheng Kung University, Tainan 70101, Taiwan; 3 Institute of Oral Medicine, College of Medicine, National Cheng Kung University, Tainan 70101, Taiwan

**Keywords:** antimicrobial properties, cytotoxicity, chitosan, chemical crosslinking, cinnamaldehyde

## Abstract

For the effective clinical antibacterial application of biomaterials, such as for wound management and tissue repair, the biomaterials need to show proper antibacterial capability as well as non-cytotoxicity. Furthermore, the material needs to have suitable mechanical characteristics for further medical use. Chitosan hydrogel is a potential candidate for various antibacterial biomedical applications due to its amine functionalities that lead to antimicrobial characteristics. Nevertheless, its antimicrobial capability is dependent upon the degree of protonation of amine groups caused by the pH value. Moreover, its mechanical compressive strength may not be high enough for clinical use if not chemically or physically crosslinked. This study utilized a novel chemical crosslinker, mercaptosuccinic acid, to improve its mechanical characteristics. The natural antibacterial agent, cinnamaldehyde, was grafted onto the crosslinked chitosan to improve its antimicrobial capability. Meanwhile, to take advantage of the thiol functionality in the mercaptosuccinic acid, the bactericidal silver nanoparticles were incorporated through silver-thiol covalent bounding. NMR analyses indicated the chitosan was successfully mercaptosuccinic acid-crosslinked and grafted with cinnamaldehyde at different ratios. Combined the results from the mechanical assessment, swelling experiments, antimicrobial assessment, and cytotoxicity assay, the chitosan hydrogel with the highest crosslinked degree and grafted with cinnamaldehyde and silver nanoparticles is of great promise for further clinical uses.

## 1. Introduction

Healthcare-associated infections (HAIs) are still great problems for both hospital workers and biomaterial researchers. Various approaches have been proposed to reduce HAIs, including the employment of new clinical management methods as well as the use of new antibacterial biomaterials [1]. The use of antibacterial material has been a research focus for developing different clinical applications such as wound dressing or urinary catheters. Besides its suitable antibacterial/bactericidal properties, the material needs to be noncytotoxic, and nonallergic, as well as proper mechanical characteristics for clinical use, such as for wound dressing application [2].

Chitosan, a linear polysaccharide composed of randomly distributed β-(1→4)-linked D-glucosamine (deacetylated unit) and N-acetyl-D-glucosamine (acetylated unit), is the second most abundant polysaccharide next to cellulose [3,4]. It has been explored for various biomedical applications, such as wound dressing, due to its good biocompatibility, antibacterial and anti-inflammatory capabilities, and hemostatic ability [5,6,7]. One of the factors that lead to its good antimicrobial capability is the electrostatic interactions between the cationic functionalities of chitosan and the microbe’s cell wall [6]. This suggested that chitosan with a higher density of cationic amine groups (i.e., a higher degree of deacetylation) would have better antibacterial properties. Nevertheless, chitosan with a high degree of deacetylation likely does not have the suitable mechanical characteristics needed for hydrogel biomedical application, even after the physical/chemical crosslinking approach to enhance its mechanical stability and integrity for clinical use. Furthermore, the swelling capability of the crosslinked chitosan hydrogel, an important characteristic that is related to the capability of absorbing the exudate, could be affected by the crosslinking methodology employed [8,9,10]. Hence, choosing a suitable crosslinking methodology is a must for creating chitosan hydrogel with needed antimicrobial properties, mechanical characteristics, and exudate absorption capability for certain clinical applications, such as wound dressing.

Cinnamaldehyde, a naturally occurring compound, and its derivatives have attracted great attention due to its substantial antimicrobial activity and different array of medicinal properties, including antitumor, anti-inflammatory, and antifungal capability [11,12]. Its antimicrobial properties have been attributed to different molecular biological mechanisms, antibiofilm formation capability, anti-quorum sensing ability, and ability to destruct microbial cell membranes [12].

Silver nanoparticles have been explored as microbicidal agents due to the surface release of silver ions, a material with a bactericidal effect against a broad spectrum of Gram-positive and Gram-negative bacteria, including several multi-drug-resistant (MDR) bacteria while not imposing severe adverse effects on humans [13,14,15]. Nevertheless, reports have indicated that the use of a dressing coated with silver nanoparticles could raise liver enzymes and argyria-like symptoms in burn patients and trigger the onset of metal allergies when tested in mice models [16,17]. This indicates that careful biological safety evaluation is needed for medical products containing silver nanoparticles.

In this investigation, a novel chemical-crosslinked chitosan hydrogel grafted with the bactericidal agents cinnamaldehyde and silver nanoparticles was explored for developing a novel antibacterial material. Mercaptosuccinic acid was utilized as a novel chemical crosslinker for chitosan. Besides improving the mechanical characteristics, the thiol functional group could enhance the silver nanoparticle attachment through silver–sulfur covalent bounding. Depending upon the nature of the bactericidal effect caused by the two different antimicrobial agents, cinnamaldehyde and silver nanoparticles, as well as the chemical synthesis formulation employed, a novel crosslinked chitosan-based hydrogel that is promising for antibacterial clinical applications was demonstrated.

## 2. Results and Discussions

**Scheme 1:**
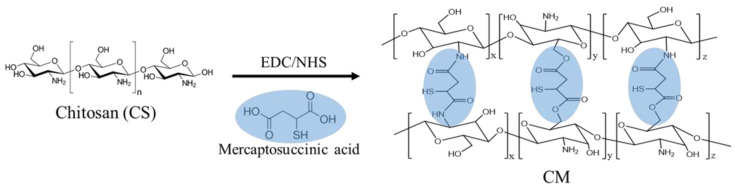

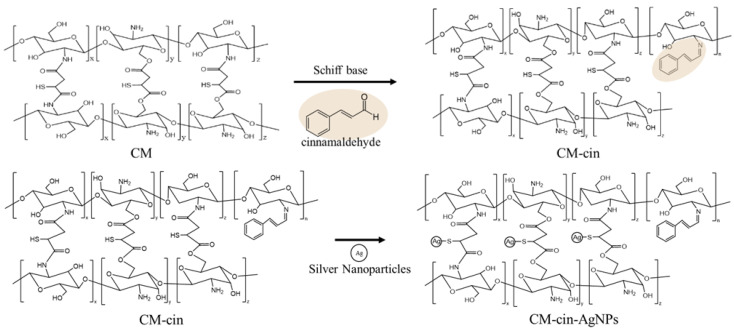
The synthesis procedure of various chitosan-based materials.

The schematic diagram for the preparation of these chitosan-based materials is shown in Scheme 1. EDC: *N*-(3-Dimethylaminopropyl)-*N*′-ethylcarbodiimide hydrochloride. NHS: *N*-hydroxysuccinimide.

### 2.1. Synthesis of Crosslinked Chitosan (CM)

**Figure 1:**
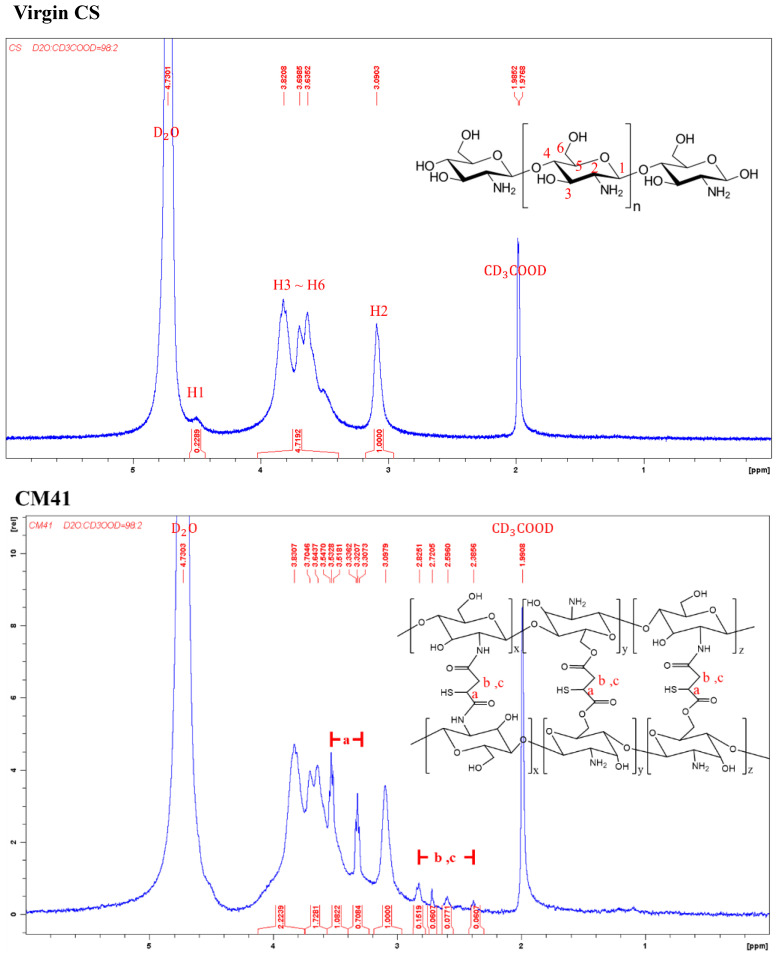

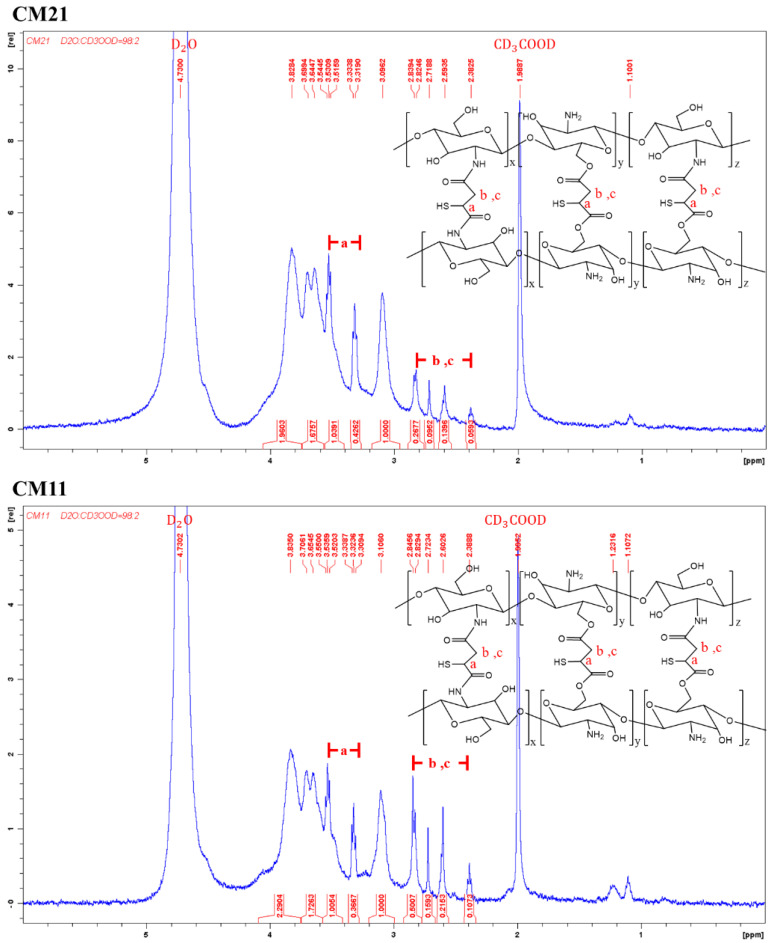
The ^1^H-NMR spectra of virgin chitosan and various crosslinked chitosan, a, b, and c are the corresponding H atoms to the indicated peaks.

The chemical structure of the virgin chitosan (CS) and various crosslinked chitosan prepared using a different weight ratio of crosslinker, mercaptosuccinic acid (MSA), to chitosan (Table 1) was analyzed by ^1^H-NMR (Figure 1). The MSA crosslinker could react with the amine or hydroxyl groups of the chitosan (Figure 1 inset). The proton peaks for the virgin CS were assigned as follows. ^1^H-NMR (500MHz, CD_3_COOD/D_2_O, 2:98 *v*/*v*). δ(ppm): 3.09 (proton at C2); 3.63–3.82 (proton at C3-C6); 4.51 (proton at C1). For the crosslinked chitosan, CM41, CM21, and CM11, the proton peaks were determined as follows: ^1^H-NMR (500MHz, CD_3_COOD/D_2_O, 2:98 *v*/*v*) δ(ppm): 1.10~1.23 (1H, -S**H**); 2.38~2.84 (2H, -C**H_2_**-); 3.30~3.33, 3.52~3.55 (1H, -C**H**(SH)-). Proton exchange between the protonic deuterated solvent, such as the D2O used here, and the proton atoms on the heteroatoms such as nitrogen, oxygen, and sulfur atoms could hinder the notion of -COOH of mercaptosuccinic acid in the CMs, whether the crosslinking reaction proceeded completely or not. The degree of crosslinking of the crosslinked chitosan was then calculated, assuming the complete reaction of carboxyl groups, by dividing the integral area of the hydrogens on MSA (δ = 2.38 − 2.84) by that of hydrogen at C2 on chitosan (δ = 3.09).

(1) $$\mathrm{Degree}\;\mathrm{of}\;\mathrm{crosslinking}\;\left(\%\right)=\frac{A_{\delta =2.38-2.84}/2}{A_{\delta =3.09}}\times 100\%$$

It was noted that with the increased amount of crosslinker, MSA, the degree of crosslinking increased from 17.5% for CM41 and 28.1% for CM21 to 49.1% for CM11.

### 2.2. Synthesis of Cinnamaldehyde Modified Crosslinked Chitosan (CM-cin)

**Figure 2:**
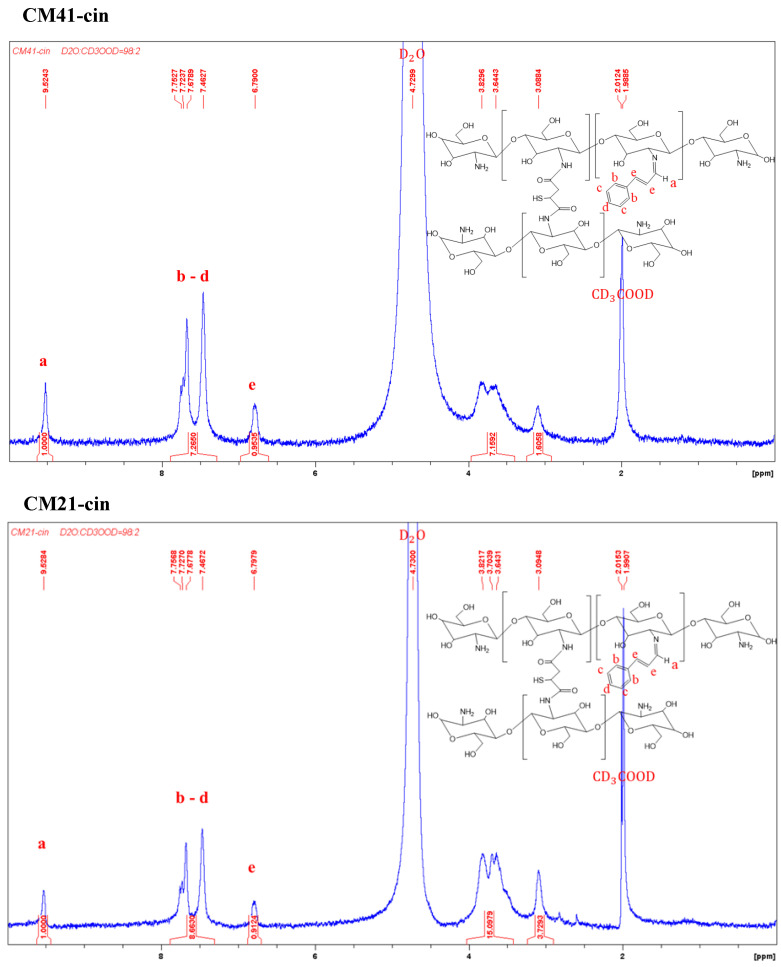

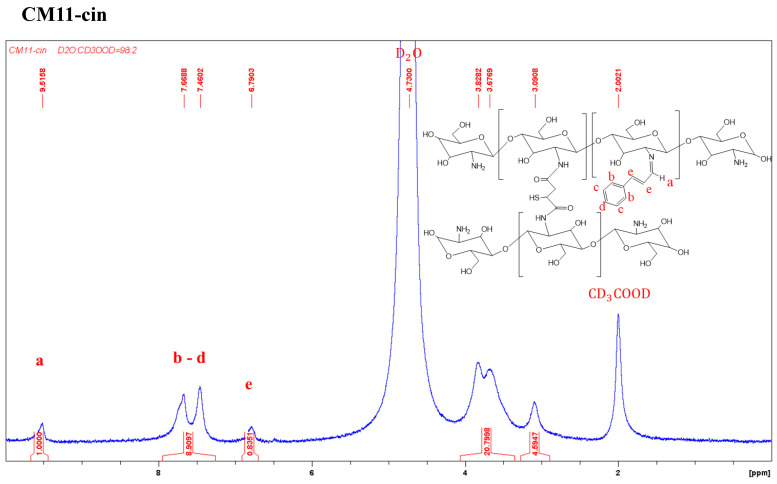
The ^1^H-NMR spectra of various cinnamaldehyde-modified crosslinked chitosan, a, b, c, d, and e are the corresponding H atoms to the indicated peaks.

The ^1^H-NMR spectra of CM41-cin, CM21-cin, and CM11-cin are shown in Figure 2. The aldehyde group of the cinnamaldehyde can react with the residual amines in crosslinked chitosan to form the imine by the Schiff-base reaction. The proton peaks of interest in this cinnamaldehyde-modified crosslinked chitosan, CM-cin, were as follows: ^1^H-NMR (500MHz, CD_3_COOD/D_2_O, 2:98 *v*/*v*); δ(ppm): 6.79 (2H, -C**H**=C**H**-); 7.46~7.75 (6H, benzene); 9.51 (1H, -C**H**=N-).

The modification ratio was defined as the ratio of chitosan monomeric unit, β-(1→4)-linked D-glucosamine, being reacted with the cinnamaldehyde, and determined by dividing the integral area of the hydrogen on imine (δ = 9.51) by that of hydrogen at C2 on chitosan (δ = 3.09).

(2) $$\mathrm{Modification}\;\mathrm{ratio}\;\left(\%\right)=\frac{A_{\delta =9.51}}{A_{\delta =3.09}}\times 100\%$$

The modification ratios of CM41-cin, CM21-cin, and CM11-cin were 62%, 27%, and 22%, respectively. This trend was consistent as expected in that, with more unreacted amine functionality in the crosslinked chitosan, a more imine functional group was formed with the same amount of cinnamaldehyde added.

### 2.3. The SEM and EDS Analyses of Various Chitosan-Based Hydrogels

**Figure 3:**
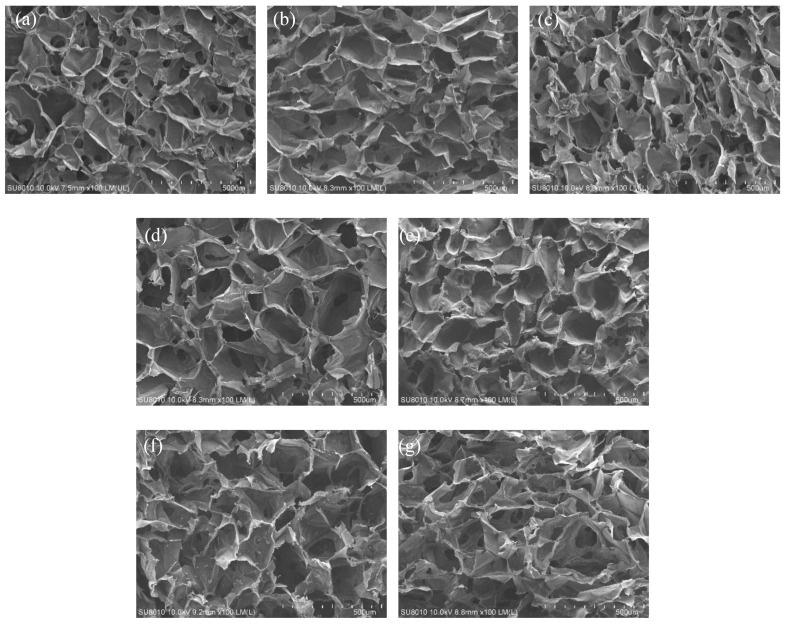
The SEM micrographs (100×) for the cross-sectional view of (**a**) Chitosan (CS), (**b**) CM41-cin, (**c**) CM21-cin, (**d**) CM11-cin, (**e**) CM41-cin-AgNPs, (**f**) CM21-cin-AgNPs, (**g**) CM11-cin-AgNPs.

The SEM micrographs of the cross-sectional view of hydrogels are shown in Figure 3. For the wound dressing application, a porous cellular structure is needed for absorbing the exudates and keeping a moist environment. The SEM micrographs revealed that, whether the hydrogels were modified with just cinnamaldehyde or both cinnamaldehyde and silver nanoparticles (AgNPs), the size and appearance of the internal pores were almost the same as those before modification, and the size of the pores was about 150–250 μm.

**Figure 4:**
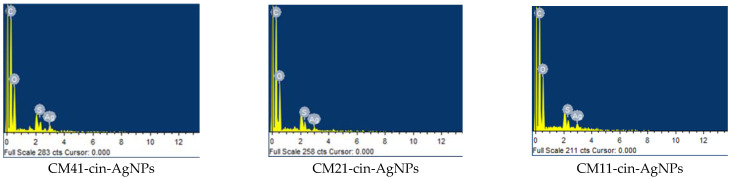
The EDS results of various AgNPs containing cinnamaldehyde-modified mercaptosuccinic acid-crosslinked chitosan hydrogels.

The EDS (energy-dispersive X-ray spectroscopy) results (Figure 4) show that even when cleaned by ultrasonication three times, a silver signal was still noted on the hydrogels incorporating AgNPs. This indicated that, by soaking the cinnamaldehyde-modified mercaptosuccinic acid crosslinked chitosan hydrogel into the AgNPs suspension, the AgNPs could be covalently bound with the thiol groups on mercaptosuccinic acid, the crosslinker, and could not be removed from the chitosan hydrogels, even by successive ultrasonic washing steps (Scheme 1). Nevertheless, it is still possible that small AgNPs could be entrapped in, not covalently bound to, the hydrogels without being removed by ultrasonic washing (see Section 2.6 antibacterial activity assessment).

### 2.4. The Swelling Characteristic of Cinnamaldehyde-Modified Crosslinked Chitosan (CM-cin) Hydrogels

A proper swelling characteristic was one of the advantages of using hydrogel in various biomedical applications, such as for wound dressings, which could not only absorb tissue fluid from the injured site but also maintain the moistness of the wound, promoting vascular proliferation and granulation of tissue formation.

**Figure 5:**
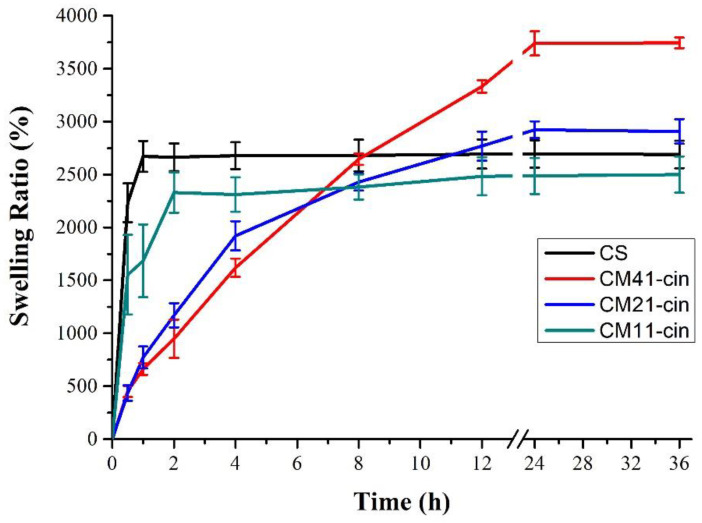
The swelling ratio of different hydrogels versus time. (*n* = 3).

In this study, the swelling ratio of different cinnamaldehyde-modified crosslinked chitosan hydrogel was analyzed after being immersed in PBS at 37 °C for different durations (Figure 5). It was noted that the initial swelling rate was decreased when more cinnamaldehyde was bound to MSA-crosslinked chitosan hydrogel (i.e., initial swelling rate: CM41-cin < CM21-cin < CM11-cin) due to the hydrophobic nature of the benzene ring in the cinnamaldehyde that led to a slower PBS infiltration to the hydrogel. The equilibrium swelling ratios were all greater than 20 times the original weight: CM41-cin (3738.6 ± 113.8%) > CM21-cin (2923.9 ± 79.8%) > CS (2694.0 ± 129.8%) > CM11-cin (2486.9 ± 170.7%). Further, a higher equilibrium swelling ratio was noted on the chitosan with a lower degree of crosslinking (see Section 2.1, synthesis of crosslinked chitosan). This may be attributed to the fact that the structure of the hydrogels prepared by this method could withstand the increment in volume expansion accompanied by liquid absorption. Furthermore, the less cross-linking agent is added, the less rigid or more flexible the internal network structure is, leading to a higher amount of PBS being absorbed.

For the non-crosslinked CS, it had a higher initial swelling ratio (<8 h) than the cinnamaldehyde-grafted crosslinked chitosan samples. This could be attributed to the hydrophobic nature of the grafted cinnamaldehyde. However, for a longer immersion duration (>24 h), the non-crosslinked CS presented a lower equilibrium swelling ratio than the CM41-cin and CM21-cin but a higher equilibrium swelling ratio than CM11-cin. The hydrogel prepared by the chitosan with less crosslinker added may allow the internal network to be more flexible and better able to accommodate the volume expansion due to PBS absorption as compared to the non-crosslinked CS hydrogel. Nevertheless, this equilibrium swelling ratio finding may be attributed to the synergistic effect caused by the degree of crosslinking and the amount of hydrophobic cinnamaldehyde grafted.

### 2.5. The Compressive Strength of Cinnamaldehyde-Modified Crosslinked Chitosan (CM-cin) Hydrogels

The compressive mechanical property test was performed by the modified ISO 3381-1, which outlines the stress and strain tests of porous material. The compressive stress was recorded at 40% strain, and the compressive modulus was then determined: CS (1.37 ± 0.15 kPa) < CM41-cin (3.32 ± 0.52 kPa) < CM21-cin (4.85 ± 0.54 kPa) < CM11-cin (7.80 ± 0.66 kPa). This indicated the compressive strength of the porous chitosan hydrogel was improved with the addition of a crosslinker, mercaptosuccinic acid. Further, with more crosslinkers added, the degree of crosslinking was increased, resulting in a great improvement in the compressive modulus.

### 2.6. Antibacterial Activity Assessment

The antibacterial activity was defined as the reduction in viable counts of Gram-positive microbe, *S. aureus* (ATCC 21351), and Gram-negative bacteria, *E. coli* (ATCC 23501) after contacting with different chitosan-based hydrogels for 6 h as compared to the control (the culture well without specimen).

**Figure 6:**
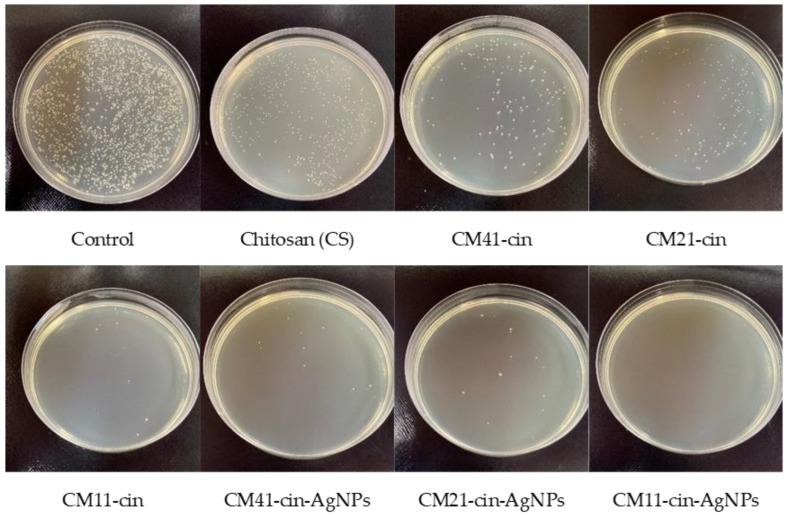
The representative agar plate image of viable *S. aureus* counts after 6 h of contact with different hydrogels.

**Figure 7:**
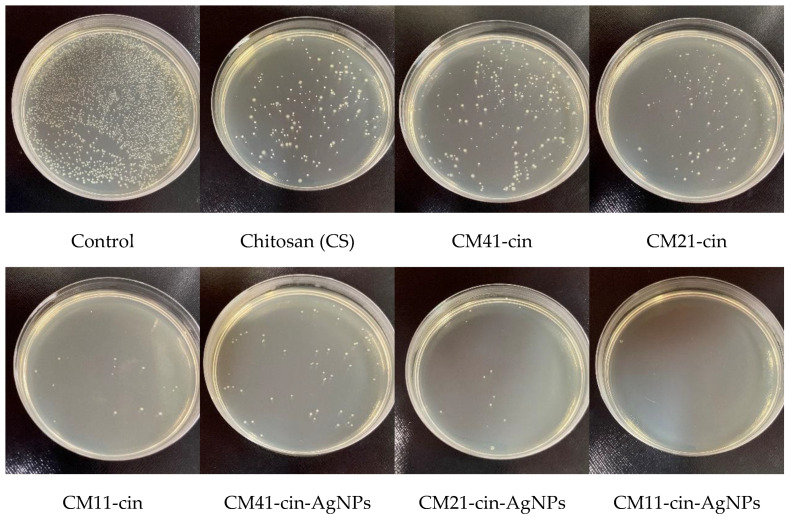
The representative agar plate image of viable *E. coli* counts after 6 h of contact with different hydrogels.

After 6 h of contact, all specimens showed a great reduction in viable microbial colony-forming units as compared to the control (Figure 6 and Figure 7, Table 2). The antibacterial activity of chitosan in *S. aureus* and *E. coli* was 90.9% and 89.5%, respectively. With the incorporation of cinnamaldehyde into the crosslinked chitosan, the antibacterial activity against *S. aureus* and *E. coli* was increased, and the antibacterial activity decreased with more cinnamaldehyde being grafted (i.e., CM41-cin < CM21-cin < CM11-cin). Previous studies [11,12] have indicated that cinnamaldehyde could impart antimicrobial capability through different “contact-initiated” bactericidal mechanisms. Despite the amount of grafted-cinnamaldehyde being higher in the order CM41-cin > CM21-cin > CM11-cin (Section 2.2), the slower absorption rate in CM41-cin in the 6 h antibacterial experiment (Figure 5) would reduce the contacting duration between the microbes and cinnamaldehyde. This may explain the lower antibacterial activity noted on the specimen with the higher amount of grafted cinnamaldehyde, CM41-cin. A similar argument could also explain the finding of the lower antibacterial activity of CM21-cin than that of CM11-cin, likely due to the slower absorption rate noted on the CM21-cin than CM11-cin (Figure 5) in the 6 h of the antibacterial assay.

With the addition of silver nanoparticles into the cinnamaldehyde-modified crosslinked chitosan hydrogels, the antibacterial activity was further improved to nearly or greater than 99%. Further, the antibacterial activity increased with the degree of crosslinking of the hydrogel (Table 2). This implies that the covalently bound AgNPs could exert a bactericidal effect such as those reported earlier [13,14,15].

The ICP-MS analyses were utilized to examine the AgNPs that were eluted after 6 h of incubation as that for the antibacterial activity assessment except for no microbes added. The concentration of silver ions released into the solution was 0.085 ± 0.020 ppm, 1.06 ± 0.03 ppm, and 4.40 ± 0.02 ppm for CM41-cin-AgNPs, CM21-cin-AgNPs, and CM11-cin-AgNPs, respectively. The release of silver increased the chance of bacteria contact with the bactericidal silver ions and further increased the antibacterial ability of the hydrogel, as noted in the antibacterial assay.

### 2.7. Cytotoxicity Assay

**Figure 8:**
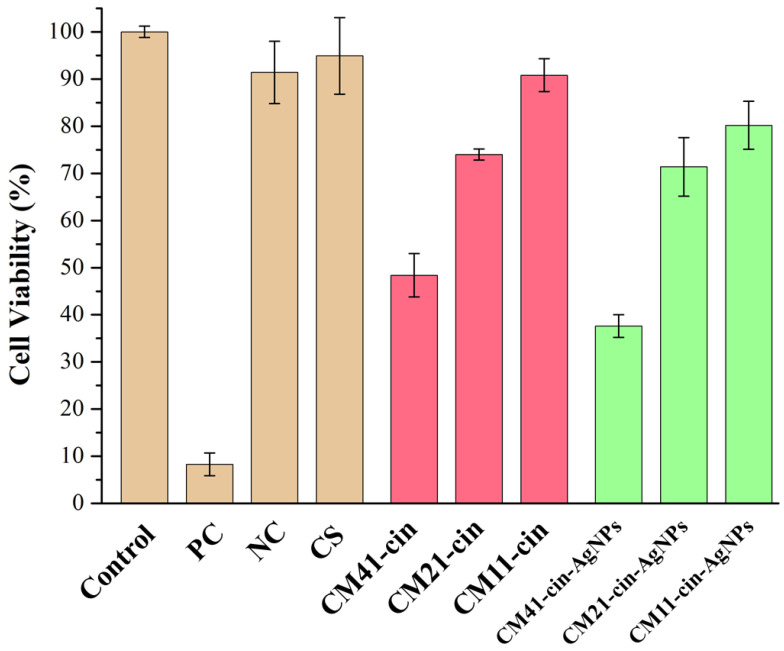
The cell viability results of the eluents from different specimens. Control: cell culture medium; PC: positive control, latex glove; NC: negative control, LDPE film (*n* = 3).

The cytotoxicity assessment results using the solution that was soaked with the hydrogel for 24 h are shown in Figure 8. According to the ISO-10993-5 standard, the sample was not significantly toxic to the cells if the cell viability was greater than 70%. It was noted the chitosan (CS) and negative control (NC; LDPE film) presented at least 90% cell viability. Further, the cell viability increased with the degree of crosslinking in both non-AgNPs- and AgNPs-containing hydrogel series (*p* < 0.05 among the samples in each series). The hydrogel with the highest degree of crosslinking in each series, CM11-cin, and CM11-cin-AgNPs, presented good cell viability at 90.8% and 80.2%, respectively. On the other hand, obvious cytotoxicity was noted in the hydrogel with the lowest degree of crosslinking in each series, the CM41-cin, and CM41-cin-AgNPs, with cell viability of 48.4% and 37.6%, respectively. This might be attributed to the likely enzymatic dissolution of small Chito-oligomeric fragments containing cinnamaldehyde and/or silver nanoparticles from the hydrogel with a lower degree of crosslinking after being immersed in the cell culture medium for 24 h.

## 3. Materials and Methods

### 3.1. Materials

Chitosan (CS, MW: 50–190 kDa, low molecular weight, 75–85% deacetylated), *N*-(3-Dimethylaminopropyl)-*N*′-ethylcarbodiimide hydrochloride (EDC), cinnamaldehyde, and sodium borohydride were purchased from Sigma-Aldrich (Milwaukee, WI, USA). Mercaptosuccinic acid (MSA) and N-hydroxysuccinimide (NHS) were purchased from Alfa Aesar (Heysham, Lancashire, UK). 2-(N-morpholino) ethanesulfonic acid (MES) was obtained from ACROS Organics (Antwerp, Belgium). Silver nitrate was purchased from BDH Laboratory Suppliers (U) Ltd. (Kampala, Uganda). Tri-Sodium citrate dihydrate (TSC) was purchased from Nacalai Tesque (Kyoto, Japan).

### 3.2. Synthesis of Crosslinked Chitosan (CM), Cinnamaldehyde Modified Crosslinked Chitosan (CM-cin), and AgNPs Containing Cinnamaldehyde-Modified Crosslinked Chitosan (CM-cin-AgNPs)-Based Hydrogels

These chitosan-base materials were synthesized as those in Scheme 1 shown above.

#### 3.2.1. Preparation of Crosslinked Chitosan (CM)

MSA, EDC, and NHS were dissolved in 10 mL MES buffer (pH 5.5) and stirred at room temperature for 30 min in the dark condition. Additionally, chitosan solution, which was prepared by dissolving 0.2 g of chitosan powder in 10 mL MES buffer, was added to the MSA/EDC/NHS solution at room temperature for 12 h in the dark condition. After the reaction, the solution was dialyzed (molecular weight cut-off, MWCO, 3000 Da) against deionized water for 48 h, and the pure products (CM41, CM21, and CM11) (Table 1) were obtained after lyophilization.

#### 3.2.2. Synthesis of Cinnamaldehyde Modified Crosslinked Chitosan (CM-cin)

A 0.1 g sample of crosslinked chitosan obtained as explained above was dissolved in 10 mL acetic acid aqueous solution (2% *v*/*v*), and 0.32 mL of cinnamaldehyde (2.54 mmol) dissolved in 5 mL ethanol was added to the crosslinked chitosan solution. The mixed solution was then reacted at 60 °C for 12 h. After the reaction, the solution was dialyzed against ethanol for 24 h and then deionized water for 48 h. Following lyophilization, the pure cinnamaldehyde-modified crosslinked chitosan (CM41-cin, CM21-cin, and CM11-cin) was obtained.

#### 3.2.3. Preparation of the CS and CM-cin-Based Hydrogels

The chitosan (CS) and cinnamaldehyde-modified crosslinked chitosan (CM41-cin, CM21-cin, and CM11-cin) were dissolved in 2% *v*/*v* acetic acid aqueous solution at the concentration of 2% *w*/*v*. The solution was then poured into 24-well plates (1 mL/well), and the plate was placed in a −20 °C freezer for 12 h. After all the liquid became solid, the plate was soaked in liquid nitrogen for 5 min, and the solvent was then removed by freeze-drying. After freeze-drying, the hydrogels were soaked in 0.1 M sodium hydroxide solution for 4 h, then washed with deionized water until the eluent became neutral. The hydrogel was further freeze-dried to remove the water.

#### 3.2.4. Preparation of the CM-cin-AgNPs Hydrogels

The AgNPs were prepared with the modified steps adapted from that by Agnihotri et al. [18]: 0.002 g of NaBH4 (0.132 mmol) and 0.052 g TSC (0.177 mmol) were mixed in 48 mL deionized water (DI water) and heated to 60 °C for 30 min in the dark with vigorous stirring to ensure a homogenous solution. At the end of the 30 min, the required volume of AgNO_3_ solution was added dropwise to the mixture, and subsequently, the temperature was further raised to 90 °C. As the temperature reached 90 °C, the pH of the solution was adjusted to 10.5 using 1 M NaOH, while heating was continued for 20 min. The suspension was allowed to cool at room temperature. The suspensions were centrifuged (12,000 rpm, 15 min) and washed thrice, followed by redispersion in DI water and stored at 4 °C. The concentration of AgNPs suspended in the water was determined by the high-resolution- inductively coupled plasma mass spectrometer (HR-ICPMS; Thermo-Element XR) operated by the Core Facility Center at National Cheng Kung University.

For the preparation of hydrogel containing AgNPs (CM-cin-AgNPs), the CM-cin-based hydrogels were immersed in 3 mL of AgNPs suspension (0.3 mg/mL) at room temperature. The AgNPs suspension was replaced with deionized water after 6 h of immersion and the unbonded AgNPs were removed by ultrasonication three times. After freeze-drying, the CM41-cin-AgNPs, CM21-cin-AgNPs, and CM11-cin-AgNPs hydrogel were obtained.

### 3.3. Characterization Methods

The chemical configuration of different crosslinked chitosan (CM) and cinnamaldehyde-modified crosslinked chitosan (CM-cin) was analyzed by nuclear magnetic resonance (NMR, Bruker AVANCE NEO 500MHz, Ettlingen, Germany). The parameters used for NMR data acquisition were as follows: number of scan: 16; delay time: 2 s; spectral width: 20 ppm; transmitter frequency offset: 6.175 ppm; acquisition time: 1.63840 s; probe temperature: 300 K; pulse: 8 μsec; power level: 21.406 W. Various techniques, including the inductively coupled plasma-mass spectrometer (ICP-MS, Thermo-Element XR, Bremen, Germany), scanning electron microscope (SEM, Hitachi SU8000, Ibaraki, Japan), and transmission electron microscope (TEM, JEOL JEM-2010) in the Core Facility Center at National Cheng Kung University were utilized to determine various morphological characteristics and silver concentration of the hydrogels prepared.

The swelling ratio (SR) of the hydrogels was determined as follows. The hydrogels were placed into a centrifuge tube containing 5 mL PBS (0.01 M pH 7.4) at 37 °C. The hydrogels were taken out from the tube, and the superficial water was removed and gently wiped off with filter paper. After that, the hydrogels were weighed and then returned to the same tube until the weight of the hydrogels did not change. SR was calculated using the equation SR = (Wt − W0)/W0, where W0 and Wt represent the initial weight and the weight at each testing time.

Compression properties of the hydrogels were determined using a 100 N load cell at a compression rate of 1 mm/min. This test was applied to wet samples in triplicate. The hydrogels were submerged in PBS for 24 h to reach saturation. The setup of the test and calculations of compressive modulus was performed by the modified ISO 3386-1 standard.

#### 3.3.1. Antibacterial Activity Assessment

*S. aureus* (ATCC 21351) and *E. coli* (ATCC 23501) were placed in Luria–Bertani broth and yeast, respectively, and cultured in an oscillating incubator at 100 rpm and 37 °C for 24 h and then diluted for use. The freeze-dried hydrogels were sterilized by immersion in 75% ethanol for 15 min and then lyophilized to remove the ethanol. Then, hydrogels were weighed and put into the 24-well plate. One milliliter of diluted bacterial solution was added to each well, and the well without hydrogel was used as the control group. The 24-well plate was placed in an oscillating incubator at 100 rpm and 37 °C. Six hours later, 100 μL of culture medium was removed and then placed onto the agar plates for 24 h incubation at 37 °C. The colonies on the agar plate were counted with a digital camera. The antibacterial activity was determined according to the following formula. Antibacterial activity (%) = (A − B)/A × 100%, where A and B were the colony-forming units of the control group (the well without specimen) and the tested sample, respectively. Values were presented as the mean ± standard deviation with *n* = 3.

#### 3.3.2. Cytotoxicity Test

The cytotoxicity of different hydrogels was assessed using the medium extracts from the hydrogels to contact the L929 cells based on the ISO 10993-5 and ISO 10993-12 standard. The Minimum Essential Medium (MEM) (Gibco) supplemented with 10% House serum (HS) (Gibco), 1% Penicillin-Streptomycin (*p*/S) (Gibco), 1% HEPES solution (Sigma-Aldrich), 1% MEM Non-essential Amino Acid Solution (100×) (Sigma-Aldrich), and 1% GlutaMAXTM-1 (100×) (Gibco) was used as the culture medium. Briefly, freeze-dried hydrogels were sterilized by immersion in 75% ethanol for 15 min, then lyophilized to remove the ethanol, and then rinsed three times by squeezing the hydrogels and immersion for 1 h with the sterilized potassium phosphate-buffered solution (PBS). Next, each sample was put into a 15 mL centrifuge tube and the culture medium was added at the volume of 1 mL medium/0.1 g sample. Next, the centrifuge tubes were incubated at 37 °C and 100 rpm for 24 h. The L929 cells were seeded in a 96-well plate at a density of 10,000 cells/well. After being cultured for 24 h, this culture medium was replaced with the 100 μL of the medium that had been used to incubate the hydrogels. Each group consisted of three replicates. After 24 h of incubation, 50 μL of MTT [3-(4.5-dimethylthiazol-2-yl-2.5-diphenyl) tetrazolium bromide] at a concentration of 1 mg/mL was added and incubated for another 2 h; the medium was then replaced with 100 μL of dimethylsulfoxide (Sigma-Aldrich) to dissolve the precipitate. The absorbance at 570 nm was measured using an ELISA reader. The reference wavelength at 650 nm.

## 4. Conclusions

NMR analyses have confirmed that the mercaptosuccinic acid crosslinking reaction and subsequent cinnamaldehyde-grafting Schiff-base reaction were successfully executed. The degree of crosslinking increased with the amount of crosslinker, mercaptosuccinic acid, added. The amount of antimicrobial cinnamaldehyde grafted is related to the amine functionality that was not consumed after the mercaptosuccinic acid crosslinking reaction. That meant the amount of cinnamaldehyde grafted in the crosslinked chitosan would decrease with the degree of crosslinking.

A proper preparation scheme has been successfully utilized to prepare the water-absorptive porous hydrogels based on the cinnamaldehyde-grafted mercaptosuccinic acid-crosslinked chitosan with or without the silver nanoparticles. The compressive strength of these porous chitosan-based hydrogels increased with the degree of crosslinking. An equilibrium water absorptive study has revealed that at least 20 times the amount of PBS can be absorbed into all of these chitosan-based hydrogels. Further, the equilibrium amount of PBS absorbed decreased with the degree of crosslinking. On the other hand, the PBS absorption rate would increase with the degree of crosslinking, likely due to the lower amount of hydrophobic cinnamaldehyde grafted in the chitosan with a higher degree of crosslinking.

The antibacterial assessment indicated that the incorporation of antibacterial cinnamaldehyde and silver nanoparticles into the crosslinked chitosan could further increase its bactericidal capability. Further, the antibacterial activity correlated with the PBS absorption rate, but not the amount of cinnamaldehyde grafted, likely due to the nature of the contact-killing characteristic associated with the cinnamaldehyde. The cytotoxicity assay using the medium soaked with the hydrogels for 24 h revealed that the specimen with the lowest degree of crosslinking was cytotoxic, likely resulting from the enzymatic dissolution of small Chito-oligomeric fragments containing antimicrobial cinnamaldehyde and/or silver nanoparticles due to the low chemical crosslinks.

Based on the mechanical property analysis, water absorption evaluation, and antibacterial and cytotoxic assessments mentioned above, the cinnamaldehyde and silver nanoparticles incorporated mercaptosuccinic acid-crosslinked chitosan with the highest degree of crosslinking is of great promise for further development in various antibacterial clinical applications, such as wound dressing. Nevertheless, the sulfhydryl group has been indicated to be oxidized by various oxidants, either in the ambient environment or inside the physiological condition [19,20,21]. Further evaluation of the stability of these cinnamaldehyde grafted and mercaptosuccinic acid crosslinked chitosan is warranted.

## Figures and Tables

**Table 1:** The chemical formulation for preparing the crosslinked chitosan, CMxy.

	Chitosan (CS)	MSA	EDC	NHS
**CM41**	0.20 g ($1.18\times 10^{-3}$ mole per monomeric unit)	0.044 g ($2.95\times 10^{-4}$ mole)	0.227 g ($1.18\times 10^{-3}$ mole)	0.271 g ($2.36\times 10^{-3}$ mole)
**CM21**	0.20 g ($1.18\times 10^{-3}$ mole per monomeric unit))	0.088 g ($5.90\times 10^{-4}$ mole)	0.453 g ($2.36\times 10^{-3}$ mole)	0.543 g ($4.72\times 10^{-3}$ mole)
**CM11**	0.20 g ($1.18\times 10^{-3}$ mole per monomeric unit))	0.177 g ($1.18\times 10^{-3}$ mole)	0.906 g ($4.72\times 10^{-3}$ mole)	1.086 g ($9.44\times 10^{-3}$ mole)

**Table 2:** The antibacterial activity of various chitosan and crosslinked chitosan hydrogels (*n* = 3).

Sample	Antibacterial Activity (%)	
	*S. aureus*	*E. coli*
	(ATCC 21351)	(ATCC 23501)
CS	90.9 ± 0.9	89.5 ± 0.6
CM41-cin	90.1 ± 3.9	93.1 ± 0.9
CM21-cin	96.2 ± 0.5	96.3 ± 0.7
CM11-cin	99.2 ± 0.1	99.4 ± 0.1
CM41-cin-AgNPs	99.4 ± 0.2	98.5 ± 0.2
CM21-cin-AgNPs	99.8 ± 0.03	99.7 ± 0.1
CM11-cin-AgNPs	>99.9	99.9 ± 0.1

## Data Availability

The data presented in this study are available on request from the corresponding author.
